# The Missing Hepatic Dimension in CKM Staging

**DOI:** 10.1016/j.jacadv.2026.102883

**Published:** 2026-06-18

**Authors:** Giovanni Ragozzino, Edi Mattera

**Affiliations:** University of Campania “Luigi Vanvitelli”, Caserta, Naples, Italy

We read with great interest the recent analysis of life expectancy across stages of the cardiovascular-kidney-metabolic (CKM) syndrome using National Health and Nutrition Examination Survey data. By linking CKM staging to absolute measures of longevity, the authors provide an important contribution to the clinical translation of this emerging construct and reinforce the central role of metabolic dysfunction in cardiovascular and renal disease progression.^1^

A methodological aspect warrants further consideration: the absence of metabolic dysfunction–associated steatotic liver disease (MASLD) from the CKM staging algorithm. MASLD is now recognized as a primary metabolic organ disease with direct cardiovascular implications. Hepatic steatosis and fibrosis amplify systemic insulin resistance, promote atherogenic dyslipidemia, sustain low-grade inflammation, and impair endothelial function—mechanistic pathways that accelerate atherosclerosis, heart failure progression, and chronic kidney disease.^2^

Importantly, MASLD and its fibrotic stages are independently associated with cardiovascular and all-cause mortality, with prognostic weight comparable to established cardiometabolic risk factors.^3^ Given its high prevalence—particularly among individuals with obesity, type 2 diabetes, and Hispanic ethnicity—excluding MASLD may introduce systematic misclassification within CKM staging. Individuals with significant steatosis or fibrosis may be assigned to early CKM stages despite harboring a risk profile aligned with more advanced disease.^4^ This misclassification likely attenuates the observed gradients in mortality and life expectancy, thereby underestimating the true burden of metabolic injury across the CKM continuum.

National Health and Nutrition Examination Survey includes several tools that could enable MASLD assessment, such as controlled attenuation parameter from transient elastography, historical ultrasound-based steatosis grading, and laboratory data enabling calculation of fibrosis scores (eg, FIB-4, NAFLD Fibrosis Score). Incorporating these measures would allow identification of individuals with clinically meaningful steatosis or fibrosis—subgroups known to exhibit disproportionately elevated cardiovascular risk.

The ethnic disparities reported in the study further underscore this point. The pronounced reduction in life expectancy among Mexican Americans is consistent with the higher prevalence and earlier onset of MASLD in this population.^4^ Without direct assessment of liver disease, part of the excess risk attributed to advanced CKM stages may reflect unmeasured hepatic burden.

In conclusion, the authors’ work represents a valuable step toward operationalizing the CKM construct. However, given the centrality of hepatic metabolic injury in cardiometabolic pathophysiology, integrating MASLD into future CKM staging is conceptually essential. A CKM framework that explicitly incorporates MASLD would more accurately reflect the multisystem architecture of metabolic dysfunction and enhance the precision of cardiovascular risk stratification.^5^

## References

[1] Ma N., Zhang X., Chou T. (2026). Cardiovascular-Kidney-Metabolic Syndrome and life expectancy in U.S. adults. JACC Adv.

[2] European Association for the Study of the Liver (EASL); European Association for the Study of Diabetes (EASD); European Association for the Study of Obesity (EASO) (2016). EASL-EASD-EASO Clinical Practice Guidelines for the management of non-alcoholic fatty liver disease. J Hepatol.

[3] Targher G., Byrne C.D., Tilg H. (2020). NAFLD and increased risk of cardiovascular disease: clinical associations, pathophysiological mechanisms and pharmacological implications. Gut.

[4] Younossi Z.M., Golabi P., de Avila L. (2019). The global epidemiology of NAFLD and NASH in patients with type 2 diabetes: a systematic review and meta-analysis. J Hepatol.

[5] Ndumele C.E., Rangaswami J., Chow S.L. (2023). Cardiovascular-kidney-metabolic health: a presidential advisory from the American Heart Association. Circulation.

